# Transcriptional control of retinal ganglion cell death after axonal injury

**DOI:** 10.1038/s41419-022-04666-3

**Published:** 2022-03-16

**Authors:** Stephanie B. Syc-Mazurek, Hongtian Stanley Yang, Olivia J. Marola, Gareth R. Howell, Richard T. Libby

**Affiliations:** 1 Department of Ophthalmology, University of Rochester Medical Center, Rochester, NY USA; 2 Medical Scientist Training Program, University of Rochester Medical Center, Rochester, NY USA; 3 The Jackson Laboratory, Bar Harbor, ME USA; 4 Cell Biology of Disease Graduate Program, University of Rochester Medical Center, Rochester, NY USA; 5 The Center for Visual Sciences, University of Rochester, Rochester, NY USA; 6 Department of Biomedical Genetics, University of Rochester Medical Center, Rochester, NY USA

**Keywords:** Neurodegenerative diseases, Molecular neuroscience

## Abstract

Injury to the axons of retinal ganglion cells (RGCs) is a key pathological event in glaucomatous neurodegeneration. The transcription factors JUN (the target of the c-Jun N-terminal kinases, JNKs) and DDIT3/CHOP (a mediator of the endoplasmic reticulum stress response) have been shown to control the majority of proapoptotic signaling after mechanical axonal injury in RGCs and in other models of neurodegeneration. The downstream transcriptional networks controlled by JUN and DDIT3, which are critical for RGC death, however, are not well defined. To determine these networks, RNA was isolated from the retinas of wild-type mice and mice deficient in *Jun*, *Ddit3*, and both *Jun* and *Ddit3* three days after mechanical optic nerve crush injury (CONC). RNA-sequencing data analysis was performed and immunohistochemistry was used to validate potential transcriptional signaling changes after axonal injury. This study identified downstream transcriptional changes after injury including both neuronal survival and proinflammatory signaling that were attenuated to differing degrees by loss of *Ddit3*, *Jun*, and *Ddit3/Jun*. These data suggest proinflammatory signaling in the retina might be secondary to activation of pro-death pathways in RGCs after acute axonal injury. These results determine the downstream transcriptional networks important for apoptotic signaling which may be important for ordering and staging the pro-degenerative signals after mechanical axonal injury.

## Introduction

Axonal injury is a critical component of many neurodegenerative diseases including glaucoma. Multiple studies show an early insult occurs to retinal ganglion cell (RGC) axons at the optic nerve head (ONH) in glaucoma that triggers signaling pathways that result in axonal degeneration and somal death [1–8]. Specifically, JUN, the canonical target of the JNKs, and CHOP/DDIT3, an important mediator of the PERK arm of endoplasmic reticulum (ER) stress signaling, are key mediators of somal apoptosis after axonal injury in RGCs. Individually, JUN and DDIT3 play important roles in apoptotic RGC death after axonal injuries including mechanical optic nerve injury and ocular hypertensive injury [9–16]. Dual deficiency of JUN and DDIT3 prevented the majority of RGC death for extended time periods after optic nerve crush (CONC)—120 days after CONC, *Ddit3/Jun* deficient retinas had ~74% improved RGC survival compared to wild-type controls [17]. In this study, *Jun*^*f*^ alleles were only recombined from ~80% of RGCs [17], suggesting these two transcription factors control nearly all proapoptotic signaling in RGCs after mechanical axonal injury. Since JUN and DDIT3’s canonical roles are as transcription factors, it is likely JUN and DDIT3 control the transcriptional nodes required for somal apoptosis after axonal injury. To date, only limited transcriptional targets of JUN and DDIT3 (i.e., ATF3, BIM) have been identified that have also been shown to be important for somal death after axonal injury. Importantly, none of these targets have been shown to phenocopy the protection to RGCs afforded by JUN and DDIT3 deficiency [10, 11, 18, 19].

Glaucomatous neurodegeneration is complex, with activation of multiple signaling pathways that may have both pro-survival and pro-degenerative roles after injury [7, 20–23]. Due to this complexity, the specific molecular cascade from inciting injury to axonal degeneration and somal apoptosis remains largely undefined. As JUN and DDIT3 are transcription factors that control the majority of proapoptotic signaling, determining the transcriptional targets of these molecules will likely identify the activation of key pro-death molecules.

Given the profound protection to RGCs after mechanical axonal injury in mice deficient in *Jun* and *Ddit3*, it is clear that these molecules are key transcriptional nodes governing axonal injury-induced RGC death. Thus, understanding the transcriptional response controlled by these genes after axonal injury will provide a detailed understanding of the molecular events that control RGC death after axonal injury. To define the transcriptional network(s) controlling RGC death after axonal injury (CONC), RNA sequencing of whole retinas was performed from wild-type mice and mice deficient in *Ddit3*, *Jun* or both *Jun* and *Ddit3*. Our results showed both neuronal and immune transcriptional networks are activated after axonal injury and that activation of these networks was differentially altered by loss of *Ddit3*, *Jun*, or *Ddit3/Jun*.

## Materials and Methods

### Mice

All experiments were approved by the University of Rochester’s Committee on Animal Resources and conducted in adherences with the Association for Research in Vision and Ophthalmology statement on the use of animals in ophthalmic and vision research. Animals received chow and water ad libitum and were housed on a 12-h light-dark cycle. Three alleles were used to generate four strains for these experiments as has been previously described [17]. Briefly, these strains include 1) germline deficient *Ddit3* mice (Jackson Laboratory, Bar Harbor, ME, Stock 005530) [referred to as *Ddit3*^−*/−*^], 2) a floxed allele of *Jun* [24] recombined with the *Six3*cre, a neural retina cre [25] [referred to as *Six3*cre^+^; *Jun*^*f/f*^ or *Jun*^*−*^^/^^*−*^], 3) mice deficient in both *Jun* and *Ddit3* [referred to as *Six3*cre^+^; *Jun*^*f/f*^; *Ddit3*^*−*^^/^^*−*^ or *Jun*^*−*^^/^^*−*^*Ddit3*^*−/−*^] and 4) wild-type control mice, such as *Six3*cre^+^; *Jun*^*+/+*^, *Six3*cre^-^; *Jun*^*f/f*^, *Six3*cre^*−*^; *Jun*^*+/f*^, *Six3*cre^*−*^; *Jun*^*+/+*^, and *Ddit3*^*+/+*^ [collectively referred to as WT]. B6.Gt(ROSA)26Sortm75.1(CAG-tdTomato*)Hze/J (TdTomato+; Jackson Laboratory, Bar Harbor, ME, Stock 025106) mice were used to assess AAV2.2-cmv-gfp-cre recombination efficiency. All mice were backcrossed at least five generations to C57BL/6 J mice. The number of animals of each genotype and treatment (determined by power analysis) was stated in Additional file 11. Experimenters were masked to genotype and condition. Mice of each genotype group were randomly selected for experimental analysis.

### Controlled optic nerve crush

Mice of 2–6 months of age were used for controlled optic nerve crush (CONC) after anesthetized with 100 mg/kg ketamine and 10 mg/kg xylazine. The optic nerve was surgically exposed and crushed just behind the globe for five seconds with self-closing forceps (Roboz RS-5027, Gaithersburg, MD) [8, 18]. Ophthalmic ointment containing neomycin, polymyxin b sulfates, and dexamethasone was applied to both eyes following the procedure (Sandoz, Princeton, NJ). Control eyes included those that underwent sham surgery (optic nerve exposed but not crushed) and those eyes that were not manipulated except receiving antibiotic ointment (naïve eyes) as optic nerve crush is known to lead to microglial alterations in the contralateral eye [26, 27]. Eyes were then harvested for RNA extraction and immunohistochemistry.

### NMDA Intravitreal injections

NMDA intravitreal injections were performed as previously described [28], Briefly, animals were anesthetized with 100 mg/kg ketamine and 10 mg/kg xylazine. The sclera was cleared with the bevel of a 33 G needle, which was then used to poke a small hole just behind the limbus. A Hamilton needle was used to deliver 2 μL of 100 mM NMDA (Sigma-Aldrich, M3262) or PBS (vehicle control). Injections were performed over the course of approximately 2 minutes to avoid sudden increases in intraocular pressure.

### RGC-specific deletion of *Jun*^*f*^ alleles with adenoassociated viral cre delivery

To generate retinas with RGC-specific deletion of *Jun*, *Jun*^*f/f*^*Ddit3*^*−*^^*/*^^*−*^ animals were bilaterally intravitreally injected with 1 μL of stock AAV2.2-cmv-gfp-cre (UNC vector core). To generate WT controls, 1 μL of AAV2.2-Cmv-cre-Gfp or AAV2.2-Cmv-Gfp (with no cre allele, UNC vector core) was intravitreally injected into the eyes of *Jun*^*+/+*^*Ddit3*^*+/+*^ animals. CONC was performed no earlier than 28 days after viral delivery to ensure sufficient recombination and endogenous protein degradation. To test recombination robustness and specificity, AAV2.2-Cmv-cre-Gfp was intravitreally injected into the eyes of Tdtomato+ animals, and Tdtomato expression was quantified for specific cell types.

### RNA sample preparation and sequencing

Dissections were completed in RNase-free conditions. Retinas were quickly dissected in cold PBS and then submerged in RNA-later (Qiagen 76106, Germantown, MD). Retinas were kept at room temperature for 24 h and then stored in RNA-later at −80 °C. RNA extraction and sequencing were performed by The Jackson Laboratory Genome Technologies Core. Retina tissues were homogenized with TRIzol (Invitrogen, Carlsbad, CA) as previously described [29]. RNA was isolated and purified using the QIAGEN miRNeasy mini extraction kit in accordance with manufacturer’s instructions. RNA quality was measured via the Bioanalyzer 2100 (Agilent Technologies, Santa Clara, CA) and poly(A) RNA-seq sequencing libraries were compiled by TruSeq RNA Sample preparation kit v2 (Illumina, San Diego, CA). Quantification was performed using qPCR (Kapa Biosystems, Wilmington, MA). RNA-seq was performed on the Illumina HiSeq4000 sequencer for 75 bp pair-end reads according to the manufacturer’s instructions.

### RNA-sequencing data analysis

RNA-sequencing data analysis were performed as previously described [29]. Briefly, fastq files of all samples were subjected to the removal of adapters and trimming low-quality bases (Phred < 30) using NGSQCToolkit v2.3 [30]. RSEM v1.2.12 was used to quantify gene expression using the trimmed reads as input [31]. RSEM internally utilizes Bowtie2 v2.2.0 as its aligner [32] with supplied annotations at default parameters against the C57BL/6 J mouse genome (mm10). Genes that had less than 1 count per million (cpm) in at least two samples were removed before normalization. Differentially expressed (DE) gene analysis was performed using Quasi-Likelihood methods in edgeR 3.20.9 package [33]. DE genes were determined comparing CONC with naïve eye group within each genotype (treatment effect) and between genotypes (interaction of treatment and genotype effect). DE genes were uploaded onto Ingenuity Pathway Analysis (IPA) software for canonical pathway, upstream regulator, disease and function, and regulatory effect analysis. Please see *statistical analysis* section for criteria of DE genes and downstream analyses in IPA.

### Immunohistochemistry

All eyes for immunohistochemistry were fixed in 4% paraformaldehyde (PFA) for 2 hours and then stored in 1 M phosphate-buffered saline (PBS) as has been previously described [9, 17]. For flat-mount staining, the anterior segment was removed and then the retina was carefully dissected from the optic cup. After PBS washes, retinas were blocked with 10% horse serum in 0.4% TritonX in PBS at 4 degrees and then incubated in primary antibodies (Additional file 12) for three overnights at 4 °C before one overnight in secondary antibodies (Alexafluor-conjugated, Invitrogen) also at 4 °C. Retinas were then washed and mounted (RGC side up) in Flurogel (Electron Microscopy Sciences, Hatfield, PA). For cryosections, the anterior segment was removed and the posterior segment was processed. After PBS washes, 14 µm cryosections were blocked with 10% horse serum in 0.1% TritonX in PBS at room temperature for several hours and then incubated in primary antibodies (Additional file 12) overnight at 4 °C. The next day cryosections were incubated with secondary antibodies (Alexafluor-conjugated, Invitrogen & JacksonImmuno, West Grove, PA) for several hours at room temperature and then counterstained with 4′,6-diamidino-2-phenylindole (DAPI, ThermoFisher, Waltham, MA) before mounting with Flurogel.

### Statistical analysis

A minimum of four retinas (both sexes mixed) were used for each experimental cohort and condition (Additional file 11). Significant DE genes resulting from treatment or treatment by genotype effect were defined with a false discovery rate (FDR) less than 0.05 [i.e. −log_10_(FDR) > 1.3] and an absolute fold change (FC) larger than 1.0. Significantly enriched canonical pathways were determined by a Benjamini-Hochberg (B-H) *p* value less than 0.05 [i.e. -log_10_(B-H p-value)>1.3]. Significantly enriched upstream regulators were defined by a Fisher exact *p* value less than 0.05 [i.e. −log_10_(*p* value) > 1.3]. Activation of a pathway or upstream regulator was determined by z-score equal or more than 2 and vice versa.

## Results

### *Jun*, *Ddit3*, and dual *Jun/Ddit3* deficiency differentially alters retinal transcriptional profiles in response to axonal injury

To characterize transcriptional changes that occur after axonal injury, whole WT, *Jun*^−*/*−^, *Ddit3*^−*/*−^, and *Jun/Ddit3*^−*/*−^ retinas were dissected for RNA sequencing three days after controlled optic nerve crush (CONC), a time point at which RGC death has just begun [18]. Principle component analysis (PCA) demonstrated the overall changes in the transcriptome between CONC and naïve conditions (DNT, did not touch) and across individual genotypes (Additional file 1a). The experimental samples were primarily separated by treatment effect (CONC vs. naïve, 9.54% variance explained) and then further modestly separated by genotype effect (WT, *Ddit3*^*−/−*^, *Jun*^−*/−*^ and *Jun*^*−/−*^*Ddit3*^*−/−*^, 8% variance explained). Differentially expressed (DE) genes were determined by comparing CONC with naïve retinas for all four experimental groups (WT, *Jun*^*−/−*^, *Ddit3*^−*/−*^, *Jun/Ddit3*^*−/*−^) using edgeR (see Methods). As expected, *Jun* and *Ddit3* were significantly elevated in WT retinas after CONC (Additional file 1b). Further, *Jun* was elevated in *Ddit3*^*−/−*^ retinas and *Ddit3* was elevated in *Jun*^−*/−*^ retinas. This was consistent with previous reports that demonstrated *Jun* deletion did not prevent DDIT3 expression in RGCs [10], and *Ddit3* deletion did not prevent JUN activation in RGCs [17], suggesting these genes did not regulate each other after optic nerve injury.

**Fig. 1:**
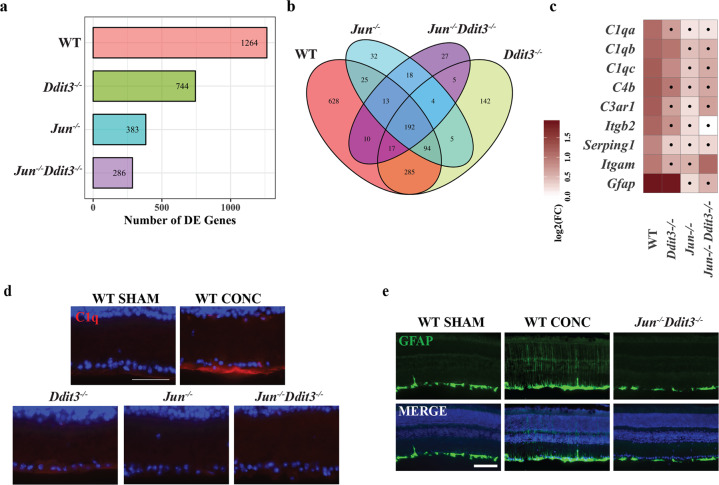
Axonal injury altered the transcription of neuroinflammatory pathways in retinal tissue. CONC led to differential expression of genes in wildtype (WT), *Ddit3* (*Ddit3*^*−/*−^)*, Jun* (*Jun*^−*/−*^*)*, and dual *Jun/Ddit3* (*Jun*^*−/−*^*Ddit3*^−*/−*^) deficient retinas (**a**). Venn diagram depicting the overlap of CONC-responding DE genes in all experimental groups (**b**). Expression of inflammatory signaling genes including complement genes and *Gfap* in WT, *Jun*, *Ddit3*, and dual *Jun/Ddit3* deficient retinas after CONC as compared to unmanipulated retinas (**c**). Area marked with a dot indicates the fold change of a gene is not significant (FDR ≥ 0.05) in a given genotype group. Area without a dot indicates the fold change of a gene is significant (FDR < 0.05) in a given genotype group. To validate the gene expression data, retinal sections were stained for C1QA and counterstained with DAPI to assess complement signaling after axonal injury. C1QA immunofluorescence was increased after axonal injury in the retinal nerve fiber layer and inner plexiform layer in WT animals after CONC injury as compared to sham retinas, however, these changes were not observed in *Jun*, *Ddit3*, or dual *Jun/Ddit3* deficient retinas after CONC (**d** *N* = 3 per genotype; Scale bar: 50μm). *Gfap* expression was also increased in WT animals but not *Jun* deficient or dual *Jun/Ddit3* deficient retinas after CONC as compared to unmanipulated retinas (**c**). Retinal sections were stained for GFAP and counterstained with DAPI after axonal injury. GFAP + presumed Müller glial processes were evident after axonal injury in WT animals as compared to sham retinas, however, these changes were not observed in dual *Jun/Ddit3* deficient retinas after CONC (**e** *N* = 3 per genotype; Scale bar: 50μm).

Overall, compared to naïve eyes, 1264 DE genes were detected in WT animals after CONC with a false discovery rate (FDR) of less than 0.05 (Fig. 1a). Retinas deficient in *Ddit3, Jun*, or *Jun/ Ddit3* had 744, 383, and 286 DE genes respectively in response to CONC. Some CONC-induced DE genes were genotype-specific while others overlapped across genotypes (Fig. 1b). Increased RGC survival correlated with fewer DE genes. Previous reports have shown deficiency in *Ddit3*, *Jun*, and *Jun/Ddit3* increased RGC survival after CONC with *Ddit3* deficiency having the least effect and *Jun/Ddit3* deficiency having the greatest effect (35 days post-CONC; WT: 21.6%, *Ddit3*: 47.5%, *Jun:* 75.4%, *Jun/Ddit3*: 88.5%) [17]. Thus, the number of DE genes resulting from injury in each genotype cohort correlated with RGC survival.

Canonical pathway analysis (Ingenuity Pathway Analysis, IPA) was employed to determine biological processes enriched in DE genes across genotypes. Analyses predicted multiple canonical pathways were modulated by CONC in WT retinas but not in retinas deficient in *Jun*, *Ddit3* and/or *Jun*/*Ddit3* (Additional file 1C, Additional file 3). These included numerous RGC-intrinsic injury signaling pathways such as ‘neuronal signaling’, ‘extrinsic inflammatory signaling’ and ‘cholesterol biosynthesis’. Compared to WT retinas, fewer pathways were significantly enriched in response to CONC in *Ddit3*, *Jun*, and dual *Jun/Ddit3* deficient animals with the dual *Jun/Ddit3* deficient animals having the fewest significantly enriched pathways. *Jun* deficiency appeared to lessen the response to axonal injury to a larger degree than *Ddit3* deficiency. It was also intriguing that apparent RGC-extrinsic proinflammatory canonical pathways were increased after axonal injury, however, these same signals were not activated in *Ddit3*, *Jun*, and dual *Jun/Ddit3* deficient retinas. These results indicate a multitude of transcriptional changes occur prior to the onset of cell death in RGCs and likely also in retinal glial cells. For instance, canonical pathway analyses predicted an attenuation of the complement cascade that has been shown to be important in neurodegeneration and to be an early extrinsic component of glaucomatous neurodegeneration [34–39]. Components of the complement cascade, including the initiating factors of the classical pathway *C1qa*, *C1qb*, and *C1qc*, were activated in WT retinas after CONC but were largely suppressed by *Ddit3*, *Jun*, and dual *Jun/Ddit3* deficiency (Fig. 1c). Additional glial genes such as *Itgam* (microglia) and *Gfap* (astrocytes, Müller glia) showed a similar pattern of expression to complement genes (Fig. 1c). To validate these findings, immunofluorescence was performed on retinal sections seven days after CONC for C1QA and GFAP. After CONC, C1Q + immunoreactivity was increased in the innermost layer of the retina near the inner limiting membrane, presumably labeling astrocytes or Müller glia endfeet as has been described in previous studies in WT retinas [36, 40], as compared to uninjured WT animals (Fig. 1d). This increase in C1Q+ immunofluorescence was markedly less in retinas from *Ddit3- Jun-*, and dual *Jun/Ddit3*-deficient mice. Similarly, *Ddit3-, Jun-*, and dual *Jun/Ddit3*-deficient retinas had less GFAP + immunoreactivity compared to WT after CONC. Immunoreactivity was primarily limited to the nerve fiber layer (NFL) and ganglion cell layer (GCL) in WT retinal sections, presumably labeling retinal astrocytes in addition to possible Müller glia endfeet (Fig. 1e). These data suggest extrinsic inflammatory responses to axonal injury in glial cells is dependent upon or mediated by intrinsic RGC signaling.

### *Jun*, *Ddit3*, and dual *Jun/Ddit3* specific signaling after axonal injury

**Fig. 2:**
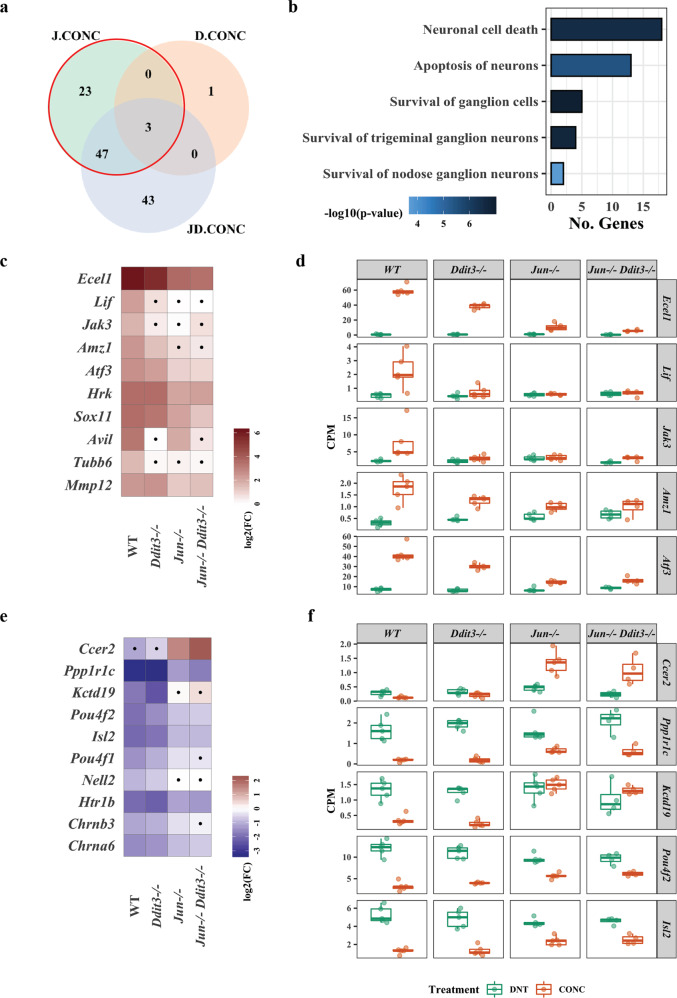
DE gene analysis revealed CONC-responding genes affected by *Jun*. The DE genes resulting from interaction of CONC treatment and genotype were determined using the following equation in edgeR package. The category of the DE genes included the CONC-responding genes dependent on *Jun* (J.CONC genes), dependent on *Ddit3* (D.CONC genes), or dependent on both *Jun* and *Ddit3* (JD.CONC). ***J*.CONC genes** = (*Jun*^−/−^ CONC – *Jun*^−/−^ DNT) – (WT CONC – WT DNT), ***D*.CONC genes** = (*Ddit3*^−/−^ CONC – *Ddit3*^−/−^ DNT) – (WT CONC – WT DNT), ***JD****.***CONC genes** = (*Jun*^−/−^*Ddit3*^−/−^ CONC – *Jun*^−/−^ *Ddit3*^−/−^ DNT) - (WT CONC – WT DNT). The Venn diagram shows the overlap of the DE genes as a result of interaction of CONC treatment and genotype, highlighting the CONC-responding genes affected by *Jun* (**a**). Cell death and survival was determined to be the top molecular and cellular function as determined by IPA analysis (shown in Table 1). The top disease and function annotations of the cell death and survival category were found to be neuronal cell death, apoptosis of neurons, and survival of ganglion cells (**b**). The fold change of top 10 CONC-responding genes activated (**c**) and inhibited (**e**) by *Jun* in all genotypes. Area marked with a dot indicates the fold change of a gene is not significant (FDR ≥ 0.05) in a given genotype group. Area without a dot indicates the fold change of a gene is significant (FDR < 0.05) in a given genotype group. Box plots demonstrate the expression levels of activated (**d**) and inhibited (**f**) genes in all genotypes after CONC (orange) as compared to unmanipulated retinas (DNT, green).

**Fig. 3:**
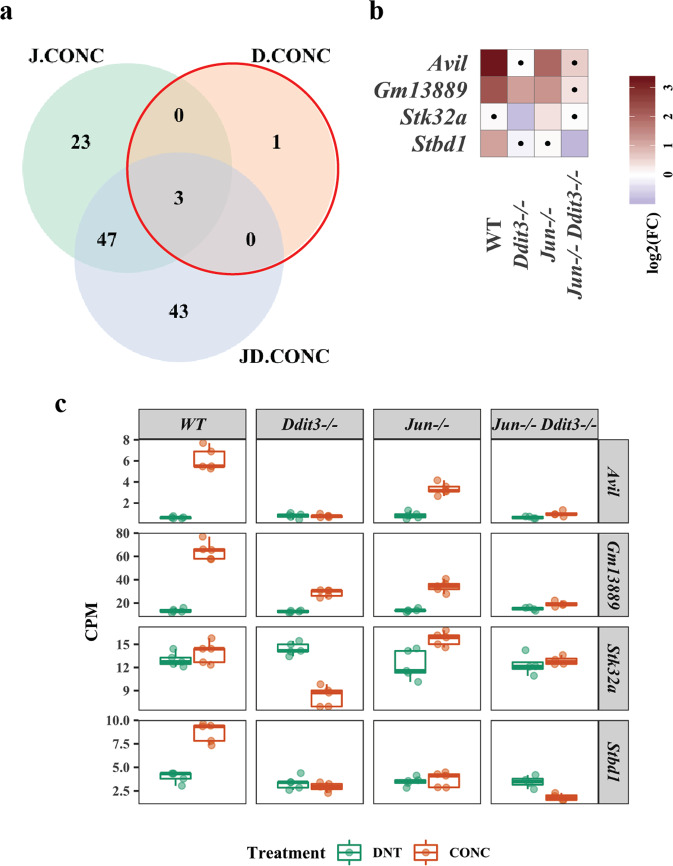
DE gene analysis revealed CONC-responding genes affected by *Ddit3*. The Venn diagram shows the overlap of the DE genes as a result of interaction of CONC treatment and genotype, highlighting the CONC-responding genes affected by *Ddit3* (**a**). The fold change of CONC-responding genes altered by *Ddit3* in all genotypes (**b**). Area marked with a dot indicates the fold change of a gene is not significant (FDR ≥ 0.05) in a given genotype group. Area without a dot indicates the fold change of a gene is significant (FDR < 0.05) in a given genotype group. Box plots demonstrate the expression levels of CONC-responding genes affected by *Ddit3* in all genotypes after CONC (orange) as compared to unmanipulated retinas (**c**, DNT, green).

**Fig. 4:**
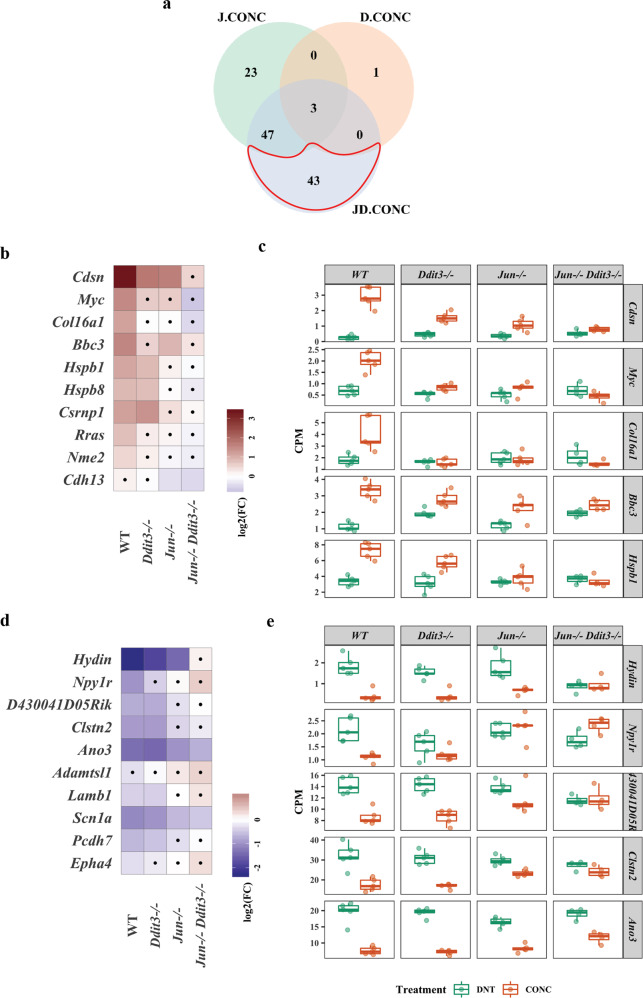
DE gene analysis revealed CONC-responding genes affected by both *Jun* and *Ddit3*. The Venn diagram shows the overlap of the DE genes as a result of interaction of CONC treatment and genotype, highlighting the CONC-responding genes affected by *Jun* and *Ddit3* together (**a**). The fold change of top 10 CONC-responding genes activated (**b**) and inhibited (**d**) by *Jun* and *Ddit3* together in all genotypes. Area marked with a dot indicates the fold change of a gene is not significant (FDR ≥ 0.05) in a given genotype group. Area without a dot indicates the fold change of a gene is significant (FDR < 0.05) in a given genotype group. Box plots demonstrate the expression levels of activated (**c**) and inhibited (**e**) genes in all genotypes after CONC (orange) as compared to unmanipulated retinas (DNT, green).

In order to determine specific *Jun*, *Ddit3*, and dual *Jun/Ddit3* targets in response to axonal injury, DE genes that were affected by the interaction of CONC treatment and gene type were determined using edgeR (FDR < 0.05, Fig. 2a). A total of 73 CONC-responding genes were predicted to be ‘regulated’ by *Jun*. Of these, 23 were unique to *Jun*, with 50 shared with *Ddit3* (47 genes) or *Jun/Ddit3* (3 genes, Fig. 2a). Enrichment of the 73 genes revealed 35 genes were related to cell death and survival including neuronal cell death, apoptosis of neurons, and survival of ganglion cells (Fig. 2b, Table 1, Additional file 5). Genes predicted to be activated by *Jun* included *Ecel1* (endothelin converting enzyme-like 1), *Lif* (leukemia inhibitory factor), *Jak3* (janus kinase 3), and known *Jun* targets *Atf3* and *Hrk* [10] (Fig. 2c,d). *Ecel1* is a member of the M13 family of endopeptidases that are important regulators of neuropeptide and peptide hormone signaling. ECEL1 (also known as DINE), has previously been shown to be activated in response to injury in both the peripheral nervous system and central nervous system– with its activation dependent upon genes including *Jun*, *Atf3, and Lif* [41]. Genes predicted to be inhibited by *Jun* included *Ccer2* (Coiled-Coil Glutamate Rich Protein 2), *Pou4f2 (*POU Class 4 Homeobox 2), and *Isl2* (SL LIM Homeobox 1, Fig. 2e, f). Only 4 CONC-responding genes were predicted to be regulated by *Ddit3*, with three of these also regulated by *Jun* (Fig. 3a). These included *Avil, Gm12889*, *Stk32a*, and *Stbd1* (Fig. 3b, c). Interestingly, 93 CONC-responding genes were predicted to be regulated by *Jun* and *Ddit3* (dual *Jun/Ddit3*). Of the 93, 43 were predicted to be regulated by a combination of both *Jun* and *Ddit3* (Fig. 4a). These included *Cdsn*, *Myc*, *Col16a1*, and *Bbc3* that were activated and *Hydin*, *Npy1r*, *Clstn2*, and *Ano3* that were inhibited by *Jun/Ddit3* (Fig. 4b, c). Collectively, this analysis reveals putative novel targets of *Jun* and *Ddit3* in axonal injury for future testing.

**Table 1:** Top 5 molecular and cellular function annotations of IPA disease and function based on 73 ONC-responding genes of affected in *Jun*^*−/−*^ mice.

Rank	Name	p-value range	# Molecules
1	Cell Death and Survival	5.54E-3–1.11E-7	35
2	Cell Morphology	5.44E-3–5.63E-6	33
3	Cellular Movement	5.54E-3–5.99E-6	31
4	Molecular Transport	4.67E-3–6.73E-6	30
5	Protein Synthesis	1.39E-3–6.73E-6	16

Each molecular and cellular function annotation contains a subset of function annotations with a p-value range and the number of involved molecules listed. Analysis revealed that 35 out of 73 DE genes are involved in cell death and survival category.

### *Ddit3, Jun*, and dual *Jun/Ddit3* deficiency attenuate glial responses after axonal injury

**Fig. 5:**
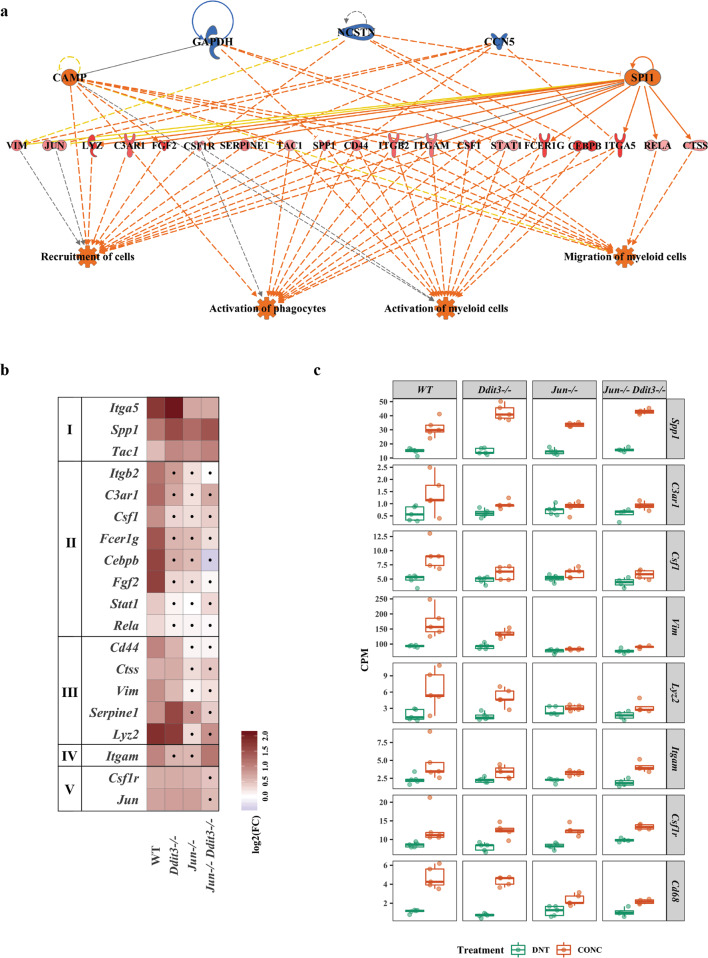
Myeloid cell function is an important upstream regulator after axonal injury. The IPA regulator effect revealed the top upstream regulator network leading to myeloid cell-related functions in WT mice (**a** red or blue color indicates the trend of activation or inhibition of an upstream regulator, target gene, or their predicted function, respectively). The top 5 upstream regulator networks among all genotypes were summarized in Table 1. The fold change of the target genes in the top regulator effect was reported for all genotypes (**b**). Genes were grouped by the significance level of the fold change observed in each strain (Group 1 gene: not affected by *Jun*, *Ddit3*, and *Jun/Ddit3* together; Group II gene: modulated by *Jun*, *Ddit3*, or *Jun/Ddit3* together; Group III: genes modulated by only *Jun*, Group IV: genes affected by either *Jun* or *Ddit3* but not *Jun/Ddit3* together; group V: genes modulated by *Jun/Ddit3* together). Area marked with a dot indicates the fold change of a gene is not significant (FDR ≥ 0.05) in a given genotype group. Area without a dot indicates the fold change of a gene is significant (FDR < 0.05) in a given genotype group. Box plots demonstrate the expression levels of selected gene related to myeloid cells functions in all genotypes after CONC (orange) as compared to unmanipulated retinas (**c** DNT, green).

IPA upstream regulator analysis was then performed to determine transcriptional hubs downstream of JUN and DDIT3 (Additional file 6a, Fig. 5). This analysis identified potentially key differences between samples deficient in *Jun* and/or *Ddit3*. For example, the upstream regulator ATF4 was largely activated in response to CONC in WT mice (Additional file 6b) but BDNF was suppressed in WT mice after injury (Additional file 6c). Regulatory effect analysis predicted a group of regulators (CAMP, GAPDH, NCSTN, CCN5) controlled a network of genes involved in activation and migration of myeloid cells and phagocytes, only in WT but not in *Jun* and/or *Ddit3* deficient groups (Table 2, Fig. 5a). Myeloid and microglia proinflammatory signaling has been thought to contribute to pathogenic signaling in neurodegenerative conditions, including after mechanical optic nerve injury [42–48]. This network contained 19 upregulated genes comparing CONC to DNT in WT retinas that were differentially suppressed by *Jun*, *Ddit3*, or dual *Jun/Ddit3* deficiency. For instance, *Itgb2*, *C3ar1*, *Csf1*, and *Stat1* were suppressed by *Jun* deficiency; *Cd44* and *Ctss* were suppressed by *Ddit3* deficiency; and *Csf1r* and *Jun* were suppressed by dual *Jun/Ddit3* deficiency (Fig. 5b, c).

**Table 2:** Top 5 predicted regulator effects.

Rank	Analysis (CONCvsDNT)	Consistency Score	Diseases & Functions	Regulator Total	Target Total
1	WT	14.224	Activation of myeloid cells, Activation of phagocytes, Migration of myeloid cells, Recruitment of cells	5	19
2	WT	14.199	Cell movement of granulocytes, Focal necrosis of liver, Invasion of cells, Migration of tumor cell lines	8	35
3	WT	12.728	Cell movement of carcinoma cell lines, Central nervous system solid tumor, Development of neuroepithelial tumor, Homing of cells, Stimulation of cells	13	50
4	WT	12.656	Formation of glomerular crescent	10	6
5	WT	12.603	Accumulation of phagocytes, Cell movement of carcinoma cell lines, Central nervous system solid tumor, Development of neuroepithelial tumor, Homing of cells, Stimulation of cells	10	51

IPA predicted a total of 100 regulator effects whose consistency score ranged from −15.011 (lowest) to 14.224 (highest) from DE genes comparing CONC vs DNT in WT, *Ddit3*^*−/−*^, *Jun*^*−/−*^, and *Jun*^*−/−*^ *Ddit3*^*−/−*^ mice.

**Fig. 6:**
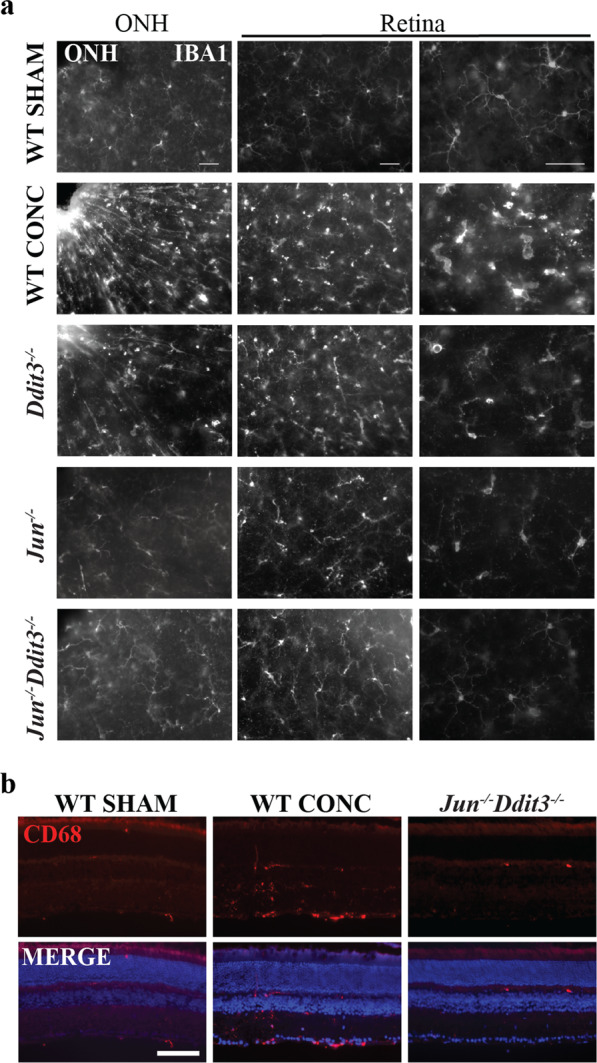
*Jun*, *Ddit3*, and *Jun*/*Ddit3* deficiency attenuated myeloid cell responses after axonal injury. Whole-mount retinas were stained with IBA1 to label microglia/macrophages 7 days after CONC. As compared to WT sham retinas, IBA + immunofluorescence was greatly increased in WT retinas after CONC at both the optic nerve head (ONH) and peripheral retina. IBA + immunofluorescence was decreased in *Ddit3* deficient animals and greatly diminished in *Jun* and dual *Jun/Ddit3* deficient retinas (**a** *N* ≥ 5 per genotype. Scale bars: 50 μm) Retinal sections were also stained for CD68 and counterstained with DAPI 7 days after CONC. Increased CD68 + immunofluorescence was evident after CONC in WT retinas as compared to sham retinas, however, these changes were not observed in the dual *Jun/Ddit3* deficient retinas after CONC (**b**, *N* = 3 per genotype; Scale bar: 50μm).

Only three genes (*Itga5*, *Spp1*, *Tac1*) remained activated in all genotypes, suggesting the majority of neuroinflammatory activity after axonal injury is mediated through *Jun* and/or *Ddit3*, but that some degree of microglial response is independent of *Jun* and/or *Ddit3*. To test this, microglia activation was evaluated using IBA1 (a marker of microglia/macrophages) and CD68 (a marker of phagocytic microglia). IBA1+ cells were assessed in whole-mount retinas from all experimental (CONC) and control (SHAM) cohorts seven days after CONC (Fig. 6a). Compared to WT sham retinas, IBA1+ immunofluorescence was greatly increased in WT retinas after CONC at both the ONH and peripheral retina. IBA1+ ‘streaks’ were apparent in WT animals after CONC that appeared to follow the path of some RGC axons as they extend from the optic nerve. In contrast to the ramified IBA1+ cells in sham retinas, amoeboid morphology was apparent after CONC in WT animals. IBA1+ immunofluorescence was noticeably less in *Ddit3*-, *Jun-* and *Jun/Ddit3*-deficient retinas compared to WT after CONC. *Jun* and *Jun/Ddit3* deficient retinas displayed a largely ramified morphology after injury, while *Ddit3* deficient animals displayed amoeboid morphology after injury. CD68 was also differentially expressed in all experimental cohorts as compared to sham control retinas of the same genotype (Fig. 5c). As compared with a paucity of CD68+ cells in sham retinas, multiple CD68+ cells were observed after CONC in the inner retina in WT retinas, with the majority observed in the GCL and IPL. CD68+ immunoreactivity was comparatively decreased in *Ddit3*, *Jun* and dual *Jun/Ddit3* deficient retinas (Fig. 6b). Together, these findings support previous reports that retinal glial activation occurs early after axonal injury and confirm transcriptional data that these proinflammatory responses are attenuated by *Jun* and *Ddit3* deficiency. Also, the level of microglial activation inversely correlated to RGC survival previously observed in each of these cohorts at extended timepoints after CONC [17].

Given *Jun/Ddit3* deletion prevented the majority of RGC apoptosis after CONC injury [17], it is likely CONC-induced glial responses are triggered by RGC-intrinsic JUN/DDIT3 activation and subsequent apoptosis. However, JUN accumulation in macroglia has been observed after glaucoma-relevant injury [49, 50] and has been implicated in driving glial activation [51, 52]. *Six3*cre has been shown to recombine floxed alleles in retinal macroglia in addition to retinal neurons [17, 25, 53]. Thus, it remained possible *Jun*/*Ddit3* deletion from glial cells resulted in attenuated glial responses after CONC.

To determine whether glial activation is triggered by RGC-intrinsic JUN/DDIT3 activation and subsequent apoptosis, or alternatively as a result of glia-intrinsic JUN/DDIT3 activation, WT and *Jun*^*−/−*^*Ddit3*^*−/−*^ animals were subjected to excitotoxic N-methyl D-aspartic acid (NMDA) injury. Unlike after CONC injury [17], *Jun/Ddit3* deletion did not prevent RGC death after NMDA injury [28]. Thus, this experimental paradigm allowed the assessment of *Jun/Ddit3*-deficient glia in response to RGC death. NMDA caused a robust increase in expression of C1Q, IBA1, CD68, and GFAP in Müller glia processes. These changes were not attenuated by glial *Jun/Ddit3* deletion (Additional file 8a-d). Likewise, increased ONH immunofluorescence of IBA1 and CD68 was not attenuated by *Jun/Ddit3* deletion from glia (Additional file 8e-g). These data suggest *Jun/Ddit3*-deficient glia are capable of activation in response to RGC death, thus suggesting attenuation of glial activation after CONC was not necessarily due to loss of glial JUN/DDIT3.

To further investigate the importance of RGC-intrinsic JUN/DDIT3 activation in mediating CONC-induced glial activation, *Jun*^*f*^ alleles were specifically recombined from RGCs using a virally delivered *Cmv*cre recombinase. As assessed with a TdTomato reporter line, AAV2.2-Cmv-cre-Gfp robustly recombined floxed alleles in RGCs, and only recombined floxed alleles in a small subset of SOX2 + Müller glia, GFAP + astrocytes, and IBA1 + microglia, and did not recombine floxed alleles in the ONH (Additional file 9). RGC-specific *Jun* deletion prevented CONC-induced increases in expression of C1Q, GFAP in Müller glia processes, IBA1, and CD68 (Additional file 10). Taken together, these data strongly implicate the importance of RGC-intrinsic JUN/DDIT3 signaling and subsequent apoptosis in driving glial activation after CONC.

## Discussion

Axonal injury is an important component of the pathogenesis of glaucoma and other neurodegenerative diseases. Unfortunately, the critical molecular signaling pathways leading from axonal injury to glaucomatous neurodegeneration are not well defined. Deficiency in the transcription factors *Jun* and *Ddit3* provides robust, long-term protection of RGC somas after axonal injury. Defining the transcriptional response controlled by these genes after axonal injury will lead to a deeper understanding of the molecular events that control axonal injury-induced RGC death, which will likely be necessary for the development of treatments for optic neuropathies [7]. However, to date, few transcriptional targets of *Jun* and *Ddit3* have been identified. To identify the downstream transcriptional targets of these molecules, whole retina RNA sequencing was performed after acute axonal injury (CONC). This approach allowed for the study of both intrinsic and extrinsic transcriptional signaling networks in retinas where intrinsic pro-death signaling had been arrested.

### JUN and DDIT3 mediated intrinsic signaling in RGCs after axonal injury

After axonal injury in the optic nerve, degeneration of the RGC soma and axon is known to be compartmentalized—that is, distinct molecular signaling pathways control axonal degeneration and somal apoptosis [1, 7, 8, 54, 55]. Transcriptional changes in the soma are also known to contribute to axonal degeneration programs after injury, including those driven by retrograde JNK-JUN signaling [56, 57].

Our study revealed *Ddit3* and *Jun* mediate activation of overlapping and distinct RGC-intrinsic processes after axonal injury. Several of these genes have been previously identified, including *Bim*, *Hrk*, and *Atf3*. Interestingly, deficiency of these genes did not phenocopy *Jun* and/or *Ddit3* deficiency after axonal injury [10, 18]. Transcriptional changes related to cell metabolism were also identified in these data, and metabolic changes are thought to play an important early role in RGC neurodegeneration [58–61]. Specific genes identified in these data that are known to be important for cellular metabolism include *Nmnat2*, *Nad*, *Sqstm1*, *Slc3a2*, and *Slc7a11*. Unfortunately, the transcriptional changes mediated by *Ddit3* and *Jun* that could be critical for controlling RGC death after axonal injury were not readily apparent in this dataset. Numerous recently published studies have assessed transcriptional changes in RGCs at the single-cell level in unmanipulated retinas and after axonal injury [62, 63]. Using bioinformatic tools to compare the data sets generated here with studies like Tran et al. [62] will be a powerful approach to unravel the critical transcriptional changes that control RGC death after axonal injury in glaucoma and perhaps other optic neuropathies.

### *Jun* and *Ddit3* deficiency uncovers extrinsic events not necessarily correlated with RGC loss

Retinal neurodegeneration after the axonal injury is complex because multiple cell types and multiple signaling pathways contribute to RGC death. *Jun/Ddit3* deficiency provides sustained, robust protection to RGCs after axonal injury, and thus these data allowed for the study of the transcriptional changes that occur in other retinal cell types in the absence of significant RGC death. *Ddit3*, *Jun*, and *Jun/Ddit3* deficiency prevented most of the neuroinflammatory signaling that occurs after CONC. For instance, both *Ddit3* and *Jun* appear to be upstream of complement activation in the retina after CONC (Figs. 1–2). This was supported by immunofluorescence showing reduced levels of C1Q in *Jun/Ddit3* deficient mice (Fig. 1). In addition, *Jun/Ddit3* deficiency appeared to lessen the presence of activated and phagocytic microglia (Fig. 6). Neuroinflammatory processes have been suggested as early extrinsic drivers of neurodegeneration in the retina [42, 48, 64, 65], but these data predict the earliest signaling after the axonal injury is intrinsic to RGCs. In accordance with this hypothesis, RGC-specific deletion of *Jun* prevented increases in C1Q, GFAP, IBA1, and CD68 immunofluorescence after CONC (Additional file 10). Thus, our data suggest RGC-intrinsic JUN activity, rather than glial JUN activity, accounts for changes in inflammatory markers after CONC. However, some immune related-genes and pathways were differentially expressed in all genotypes after optic nerve crush. These included the myeloid-related transcription factor *Spp1*, a gene which we have shown is important in neuroinflammatory responses [29]. These data show some extrinsic processes could not be prevented by deletion of *Jun/Ddit3*, highlighting the need for further studies exploring the relationship between RGC pro-death factors and extrinsic neuroinflammatory processes. Future work should consider single-cell sequencing of glaucoma-relevant cell types to improve the resolution of the molecular control of RGC death.

### Limitations of this study and future directions

A limitation to our experimental approach is the *Ddit3* allele is a germline deletion, while the *Jun* allele is a floxed allele, which was recombined with the inner neural retinal cre *Six3*cre. *Six3*cre is known to recombine alleles in astrocytes and likely Müller glia, as it is expressed in early retinal progenitor cells [25, 53]. Thus, it is possible the RGC protection afforded by this model of *Jun* and *Ddit3* deficiency and the transcriptional changes that occur in each experimental cohort after CONC may be confounded by the manipulation of signaling pathways in retinal cells other than RGCs. One example of this possibility is that intravitreal injection of TNF activates JUN in Müller glia. Intravitreal injection of TNF prior to CONC was protective of RGCs, and the mechanism for this protection has been proposed to be JUN activity in Müller glia [66]. Given retinal injury may activate JUN in other cell types, which might serve protective or deleterious roles, it is important to interpret whole retina transcriptional changes within these limitations. Another factor that might confound the analysis of transcriptional data is the recombination efficiency of *Six3c*re (known to recombine *Jun* floxed alleles in ~80% of RGCs) [9, 17]. Given this, it is possible the number of significantly regulated genes in *Jun* and *Ddit3/Jun* deficient retinas may represent some genes which are strongly expressed in cells that continue to express JUN. Thus, the number of differentially regulated genes may in fact be lower than reported in *Jun* and *Ddit3/Jun* deficient retinas. Finally, we have previously shown an uncoupling of the transcriptional response in the retina and ONH in glaucoma. In this study, we did not profile the ONH where changes to supporting cells, such as astrocytes, microglia, and vascular cells, such as endothelial cells and pericytes may be similarly important in determining axonal degeneration or survival.

## Conclusions

Transcriptional changes after axonal injury are an important part of the signaling cascade driving RGC death. We identified the transcriptional changes that occur downstream of *Jun* and *Ddit3*, two transcription factors that are known to control the majority of axonal injury-induced RGC death. By specifically identifying the transcriptional changes that occur in retinas where intrinsic pro-death signaling has been arrested, these data also allowed for the study of RGC extrinsic signaling changes that occur after axonal injury. We determined neuronal and proinflammatory signaling is significantly upregulated after axonal injury; *Ddit3*, *Jun*, and *Jun/Ddit3* deficiency attenuate differentially expressed genes after injury; and finally, proinflammatory changes after acute axonal injury in the retina are likely secondary to RGC apoptosis rather than a primary driver of retinal neurodegeneration. These results identify downstream networks of *Jun* and *Ddit3* that may be broadly important for neurodegeneration.

## Supplementary information

Supplemental Material

Reproducibility Checklist

Author Contribution Form

Gene lists comparing between CONC and DNT within all genotypes (treatment effect).

The complete canonical pathways for each genotype from Ingenuity Analysis Pathway

Gene lists comparing CONC and DNT between genotypes (treatment by genotype effect).

Complete list of the top Diseases and Functions Annotation enriched from J.CONC gene under the Cell Death and Survival category

Complete list of upstream regulators for each genotype from Ingenuity Analysis Pathway

## Data Availability

The dataset(s) supporting the conclusions of this article is(are) included within the article (and its additional file(s)). The sequencing dataset discussed in this publication is deposited at NCBI’s Gene Expression Omnibus (GEO accession number GSE168789).
