# Supplementary material for: Transcriptional control of retinal ganglion cell death after axonal injury
Source: Cell Death Dis. 2022 Mar 16;13(3):244. doi: 10.1038/s41419-022-04666-3 (PMC8927149; doi:10.1038/s41419-022-04666-3)
Supplement: Supplementary file 1 — Supplemental Material [file 41419_2022_4666_MOESM1_ESM.pdf]

Supplementary File Information

Additional file 1 *Jun*, *Ddit3*, and dual *Jun/Ddit3* deficiency alters transcription in response to axonal injury

Principal component analysis (PCA) plot showing the second and third component of transcriptional expression profiles of CONC as compared to unmanipulated retinas [did not touch (DNT)] of wildtype (WT), *Ddit3* (*Ddit3*$^{-/-}$), *Jun* (*Jun*$^{-/-}$), and dual *Jun/Ddit3* (*Jun*$^{-/-}$*Ddit3*$^{-/-}$) deficient retinas (**a**). The expression levels of *Ddit3* and *Jun* in WT, *Ddit3*, *Jun*, and *Jun/Ddit3* deficient mice (**b**). *Ddit3* and *Jun* were elevated in WT mice and *Jun* was elevated in *Ddit3* deficient retinas and similarly, *Ddit3* was elevated in *Jun* retina suggesting these gene are independently regulated. CONC-related canonical pathways were evaluated in WT, *Ddit3*, *Jun*, and *Jun/Ddit3* deficient mice (**c**). Area marked with a dot indicates a pathway is not significantly enriched (B-H p-value ≥ 0.05) a given genotype group. Area without a dot indicates a pathway is significantly enriched (B-H p-value < 0.05) in a given genotype group. Multiple canonical pathways were differentially regulated after axonal injury including neuronal signaling, inflammatory signaling, and cholesterol biosynthesis.

Additional file 2 Gene lists comparing between CONC and DNT within all genotypes (treatment effect).

The excel sheet includes gene lists resulting from comparisons between CONC and DNT within each genotype (WT, *Ddit3*, *Jun*, and *Jun/Ddit3*). Significant DE genes were determined by

FDR<0.05. Positive fold change (FC) value means the gene expression is increased in CONC compared to DNT and vice versa.

Additional file 3 The complete canonical pathways for each genotype from Ingenuity Analysis Pathway

The canonical pathways were enriched from DE genes comparing CONC and DNT within each genotype.

Additional file 4 Gene lists comparing CONC and DNT between genotypes (treatment by genotype effect).

The excel sheet includes gene lists resulting from comparisons between CONC and DNT between genotypes. The formulas illustrating the comparisons were detailed in Fig. 2 legends. Significant DE genes were determined by FDR<0.05. Positive fold change (FC) value means the gene expression is increased in *Ddit3*, *Jun*, and *Jun/Ddit3* deficient mice in response to CONC compared to WT mice and vice versa.

Additional file 5 Complete list of the top Diseases and Functions Annotation enriched from J.CONC gene under the Cell Death and Survival category

Additional file 6 *Jun* and *Ddit3* deficiency partially affect CONC induced upstream regulators responses

Top upstream regulators responding to CONC in WT, *Ddit3*, *Jun*, and *Jun/Ddit3* deficient retinas (**a**). Positive z-sore (red) indicates activation of an upstream regulator, negative z-sore (purple) indicates inhibition of an upstream regulator. Area with dot means the upstream regulator is not significantly activated or inhibited in a given genotype ($-2 < \text{z-score} < 2$), the area without a dot means the upstream regulator is significantly activated ($\text{z-score} \geq 2$) or inhibited ($\text{z-score} \leq 2$) in a given genotype. The ATF4 controlled network was an example of a network that was activated in WT mice after CONC (**b**). Red and green indicates the trend of activation or inhibition of the downstream targets of ATF4, respectively. The BDNF controlled network was an example of a network that was inhibited in response to CONC in WT mice (**c**).

Additional file 7 Complete list of upstream regulators for each genotype from Ingenuity Analysis Pathway

The upstream regulators were enriched from DE genes comparing CONC and DNT within each genotype.

Additional file 8 *Jun/Ddit3* deficiency did not attenuate glial responses after NMDA injury.

Retinal and optic nerve head sections from WT and $Jun^{-/-}Ddit3^{-/-}$ mice 3 days after intravitreal NMDA. In WT retinas, NMDA insult caused robust increases in C1Q expression (**a**), GFAP expression in Müller glia processes (**b**), IBA1 immunofluorescence (**c**) and CD68 expression (**d**).

No attenuation of glial responses was observed in *Jun*$^{-/-}$*Ddit3*$^{-/-}$ retinas after NMDA insult. Increased IBA1 immunofluorescence and CD68 expression in the optic nerve head was also not prevented by *Jun* and *Ddit3* dual deletion after NMDA injury (**d-f**) (N=3 per genotype per condition; Scale bars: 50 μm).

Additional File 9 AAV2.2-cmv-gfp-cre recombined floxed alleles robustly and specifically in RGCs

Retinal and optic nerve head sections depicting retinas from Tdtomato^{+} mice transfected with intravitreally delivered AAV2.2-cmv-gfp-cre. AAV2.2-cmv-gfp-cre recombined floxed alleles (as evidenced by Tdtomato expression) in 91.7±4.1% RGCs, 29.5±4.4% SOX2+Müller glia, 6.6±1.8% retinal GFAP+ astrocytes, and 1.8±1.2% IBA1+ myeloid cells. Arrows indicate examples of TdTomato+ cell types of interest, arrow heads indicate examples of TdTomato- cell types of interest. Of note, no TdTomato expression was observed in optic nerve cells (N=6, scale bar: 50μm).

Additional File 10 RGC-specific recombination of *Jun*^{fl} alleles with AAV2.2-cmv-gfp-cre prevented CONC-induced glial activation

Retinal and optic nerve head sections from WT and *Jun*$^{fl/fl}$*Ddit3*$^{-/-}$ mice treated with AAV2.2-cmv-gfp-cre 7 days after CONC. In WT retinas, CONC caused an increase in C1q immunofluorescence (**a**), GFAP expression in Müller glia processes (**b**), IBA1 immunofluorescence (**c**), and increased expression of CD68 (**d**). These changes were not observed in retinas with RGC-specific *Jun*

deletion. In WT retinas, CONC induced a robust increase in IBA1 immunofluorescence (**e, g**) and CD68 expression (**f, g**), however, these changes were not observed with RGC-specific *Jun* deletion (N≥3 per genotype per condition; Scale bars: 50μm).

Additional file 11 Table of animal information

Additional file 12 Table of antibodies

Additional file 1

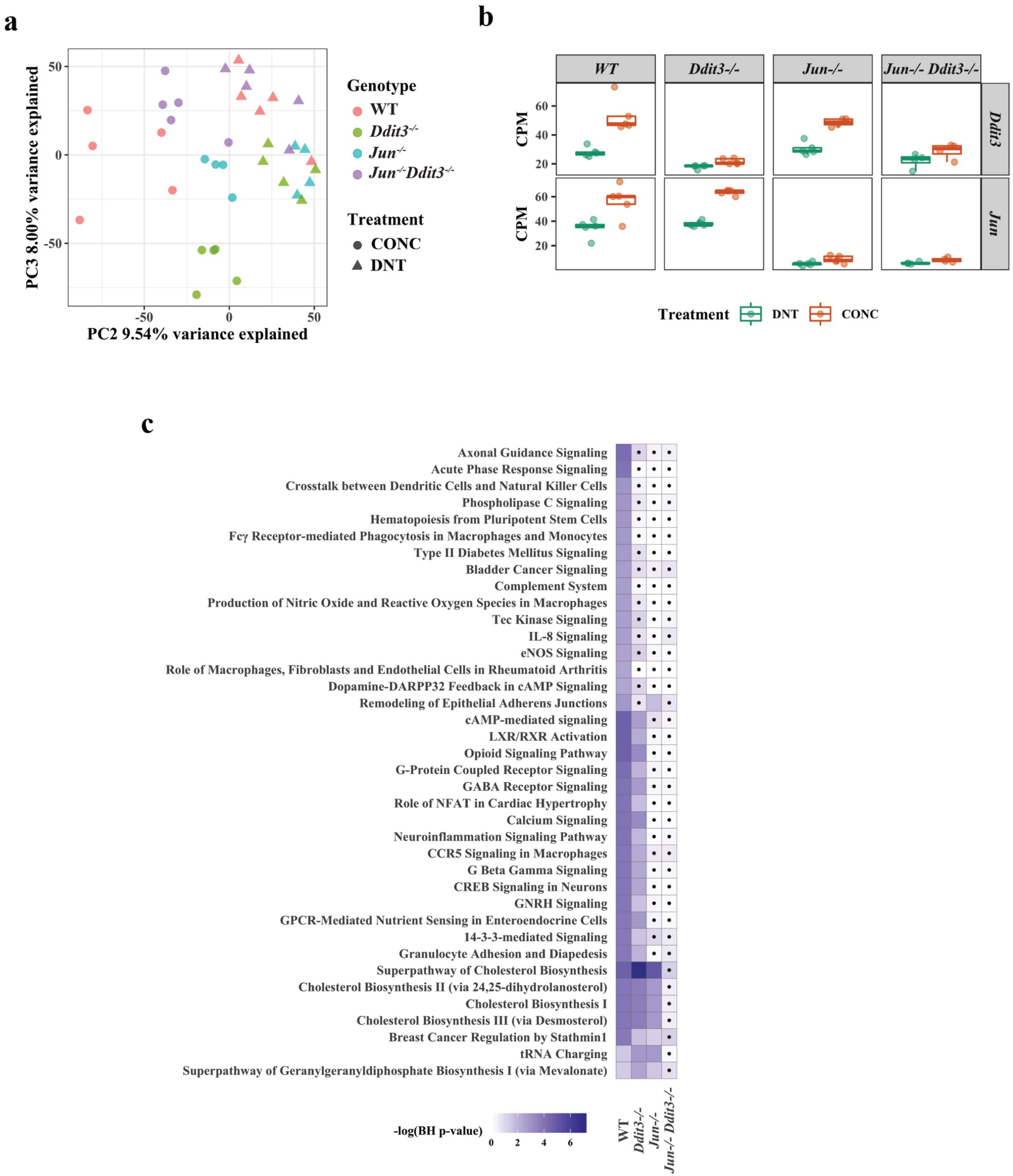

Additional file 6

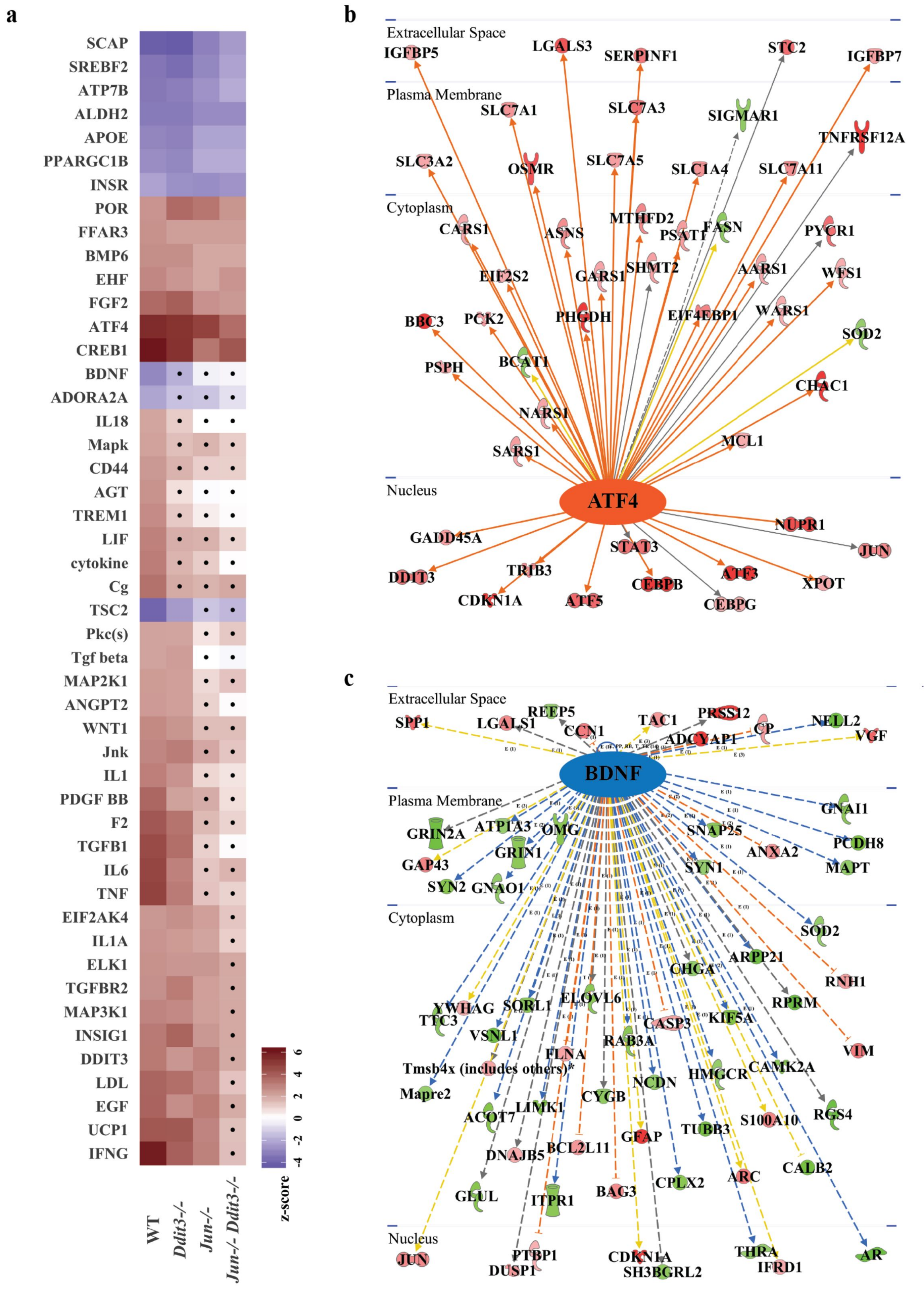

Additional file 8

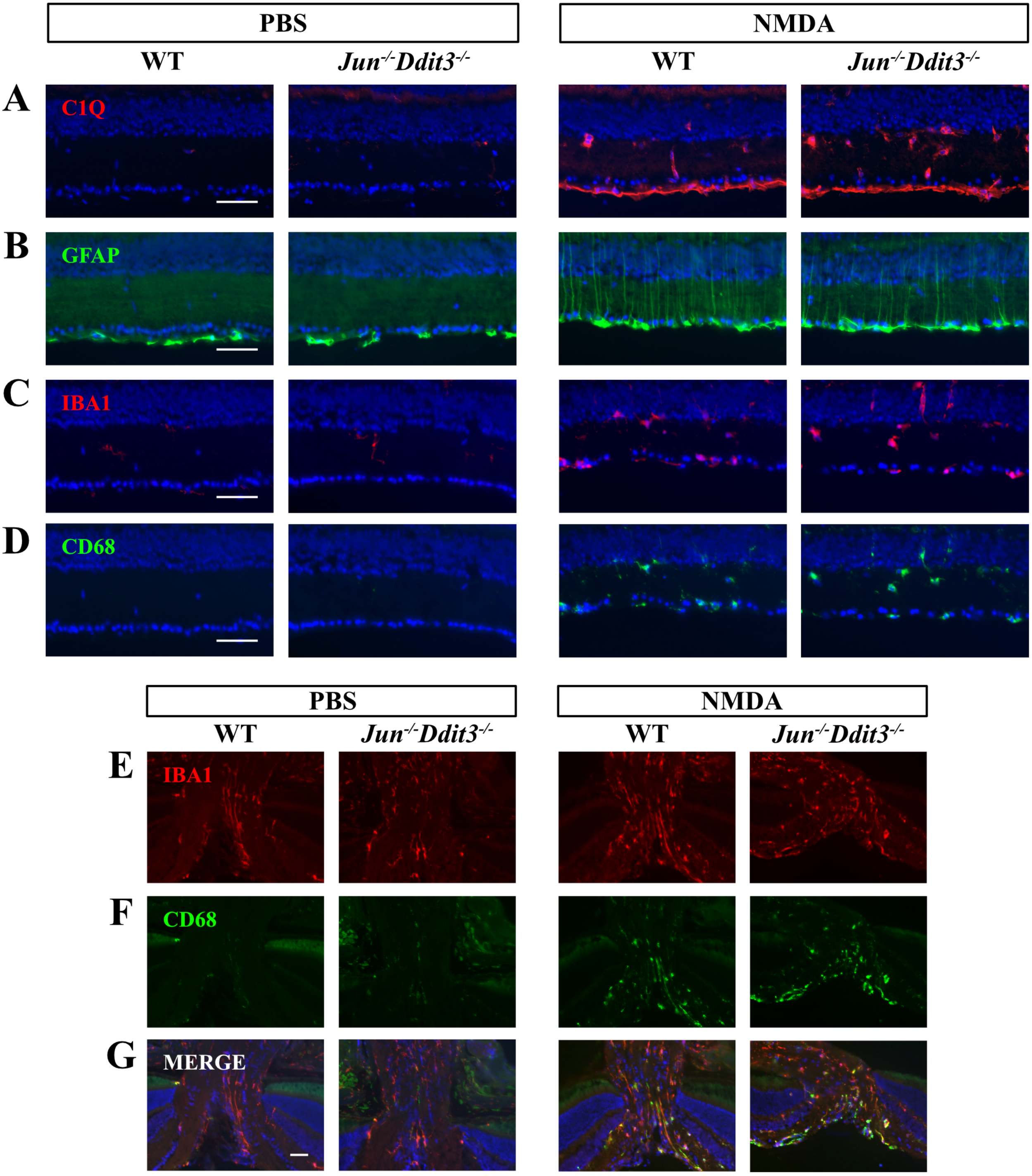

Additional file 9

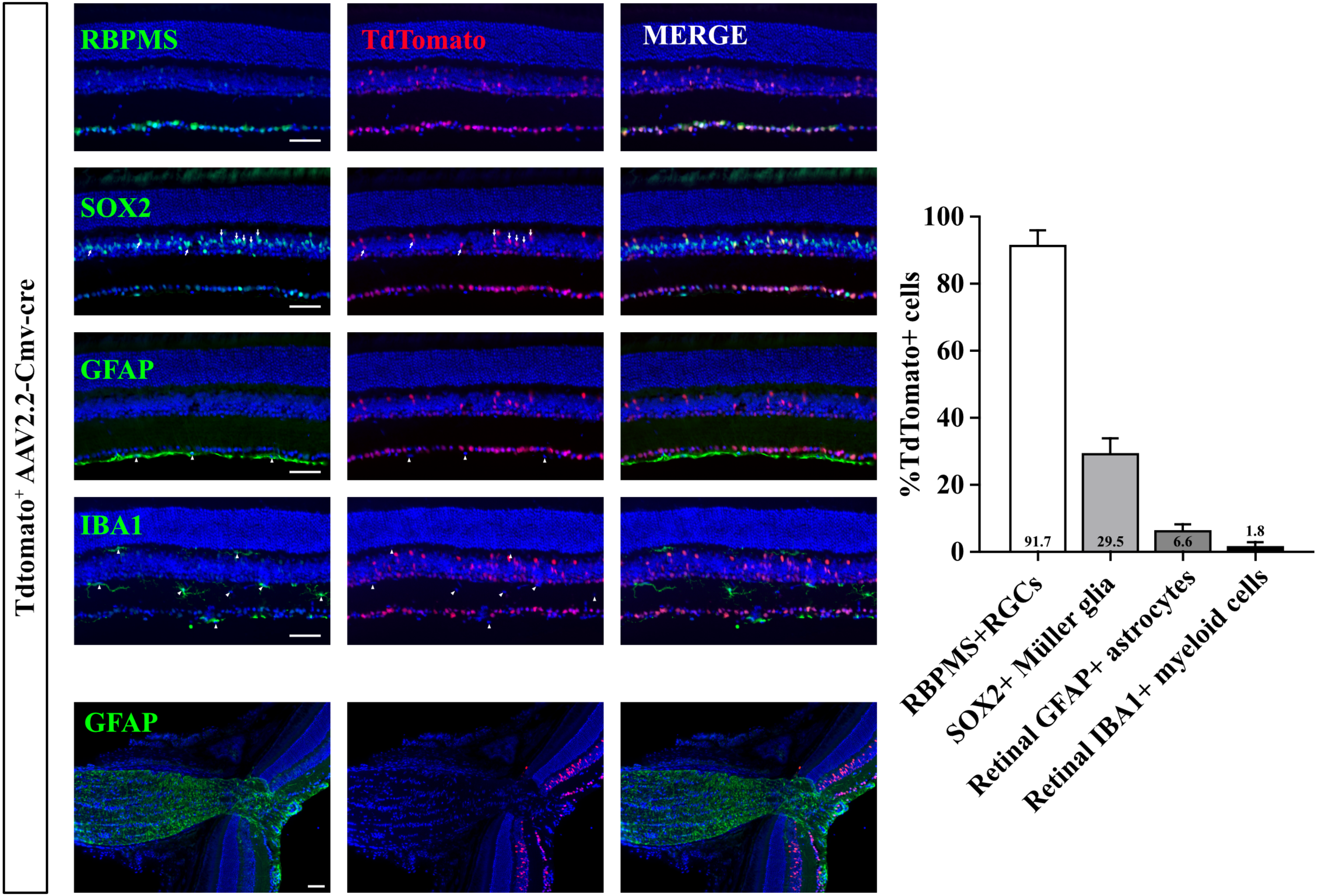

Additional file 10

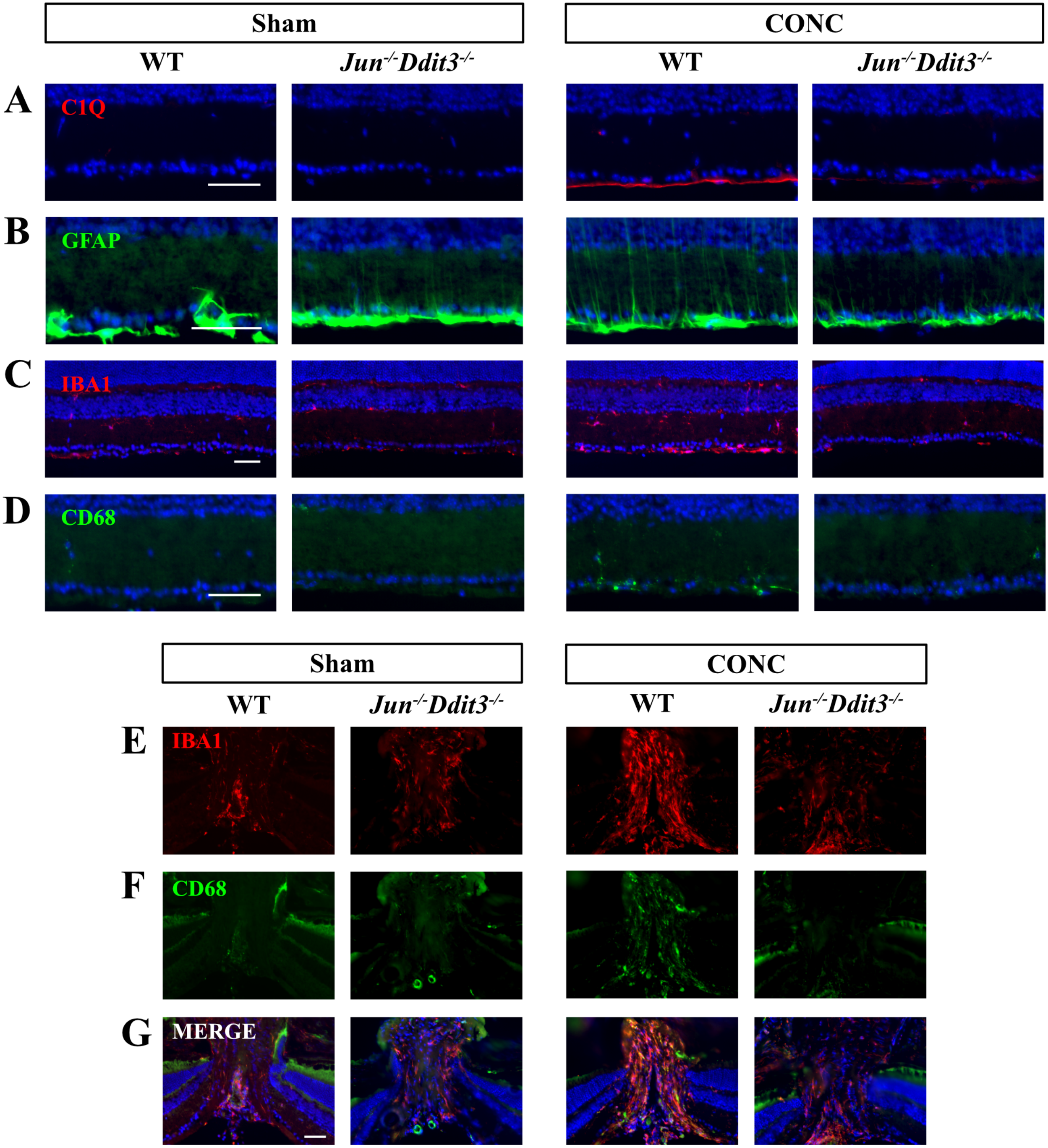

Additional File 11

Group	Sex	N
WT.CONC	F	3
WT.CONC	M	2
WT.DNT	F	3
WT.DNT	M	2
Ddit3 $^{-/-}$-Jun $^{-/-}$.CONC	F	4
Ddit3 $^{-/-}$-Jun $^{-/-}$.DNT	F	4
Ddit3 $^{-/-}$.CONC	F	1
Ddit3 $^{-/-}$.CONC	M	4
Ddit3 $^{-/-}$.DNT	F	1
Ddit3 $^{-/-}$.DNT	M	4
Jun $^{-/-}$.CONC	F	3
Jun $^{-/-}$.CONC	M	2
Jun $^{-/-}$.DNT	F	3
Jun $^{-/-}$.DNT	M	2

Additional file 12

	Primary Antibody (catalog #)	Antibody Concentration	Company
Immunohistochemistry			
CD68	Monoclonal rat anti-CD68 (MCA1957)	1:1000	BioRad
C1q	Monoclonal rabbit anti-C1q (ab182451)	1:200	Abcam
GFAP	Polyclonal chicken anti-GFAP (ab4674)	1:1000	Abcam
IBA1	Polyclonal goat anti-IBA1 (ab5076)	1:1000	Abcam
RBPMS	Polyclonalrabbitanti-RBPMS (GTX118619)	1:250	Genetex
SOX2	Polyclonal goat anti-SOX2 (sc-17320)	1:200	SantaCruz
